# Vertical Transport Control of Electrical Charge Carriers in Insulator/Oxide Semiconductor Hetero-structure

**DOI:** 10.1038/s41598-018-23990-3

**Published:** 2018-04-04

**Authors:** Jinwon Lee, Kapsoo Yoon, Keon-Hee Lim, Jun-Woo Park, Donggun Lee, Nam-Kwang Cho, Youn Sang Kim

**Affiliations:** 1 Samsung Display Company, Ltd, 181 Samsung-ro, Tangjeong-myeon, Asan-si, Chungcheongnam-Do, Republic of Korea; 2 Program in Nano Science and Technology, Graduate School of Convergence Science and Technology, Seoul National University, 1 Gwanak-ro, Gwanak-gu, Seoul, 08826 Republic of Korea; 3 Department of Physics, Hanyang University, 222 Wangsimni-ro, Seongdong-gu, Seoul, 04763 Republic of Korea; 4 Advanced Institute of Convergence Technology, 145 Gwanggyo-ro, Yeongtong-gu, Suwon, 16229 Republic of Korea

## Abstract

The technology for electrical current passing through an insulator thin-film between two electrodes is newly getting spotlights for substantial potentials toward advanced functional devices including a diode and a resistive switching device. However, depending on an electrode-limited conduction mechanisms of the conventional devices, a narrow processing window for a thickness of the insulator thin-film and an inability to control a magnitude and direction of the currents are challenges to overcome. Herein, we report a new approach to enable electrical charge carriers to pass stably through a relatively-thick insulator layer and to control a magnitude and polarity of the currents by applying an oxide semiconductor electrode in a metal/insulator/metal structure. We reveal that the electrical conduction in our devices follows a space charge-limited conduction mechanism which mainly depends on the charge carriers injected from contacts. Therefore, characteristics of the current including a current value and a rectification ratio of input signal are precisely controlled by electrical properties of the oxide semiconductor electrode. The unique current characteristics in metal/insulator/oxide semiconductor structures give extendable inspirations in electronic materials science, even a prominent solution for various technology areas of electronics.

## Introduction

Transferring electrical charge carriers directly through an electrical insulator thin-film is a prominent and classical subject for various electronic devices including metal/insulator/metal (MIM) diodes and resistive switching (RS) memories^1–4^. However, most electrical insulator thin-films have small electron affinity values and large energy band gaps, which causes to form a large energy barrier at the junction with semiconductor or metal contacts^5^. The energy offset at the junctions prevents the charge carriers from flowing between contacts, thereby blocking the flow of electrical current through the insulator layer under the operating electric field. On the other hand, when a larger electric field is applied to the insulator layer, permanent damage due to a dielectric breakdown occurs and the device performance is lost. Therefore, in MIM diodes or RS devices, electrical charge carriers are transported by other conduction mechanisms than band conduction. The conduction of electrical charge carriers in MIM diodes by a quantum tunnel effect enables electrons to pass almost instantaneously through a narrowed barrier of insulator film to the opposite electrode^6^. In the case of RS devices, a conductive path for electrical charge carriers should be formed inside the insulator layer by an external forming electric field, and resistance changes of the conductive path by set and reset voltages induce the memory characteristics^7,8^. These conduction mechanisms depend mainly on the electrical properties at the interface between an insulator/electrode contact, so these are called an electrode-limited conduction mechanism. The energy barrier height at the interface of insulator/electrode junction is a key parameter in the electrode-limited conduction mechanisms. Depending on the conduction mechanisms of MIM and RS devices, thickness of the insulator film in the devices has been limited to a few tens of nanometres or less, which narrows the processing window of the insulator films. And it has been difficult to control the direction and magnitude of the electrical currents controlled by the electrode-limited conduction mechanisms in conventional devices^9,10^.

Meanwhile, there are other conduction mechanisms through insulator layers that depend on the electrical properties of the insulating film itself, which are classified as bulk-limited conduction mechanisms. The bulk-limited conductions are dominantly controlled by trap states and trap energy levels in insulator materials, and most types of leakage currents in various devices including a field effect transistor (FET) and a metal/insulator/semiconductor (MIS) junction are dependent on the bulk-limited conduction mechanisms^11^. Recent studies on the relationship between a defect density in dielectric materials and a leakage current have revealed that the dielectric defect density at a contact junction correlates directly with the leakage current through the dielectric film^12,13^. It highlights that conventional insulating films can become to transport stably electrical charge carriers without an electrical breakdown by controlling contact junctions or defect densities in the insulator layers. Precise control of the origins and densities of defect states in insulator thin-films has been a challenging technique. Therefore, it can be an efficient approach to control the bulk-limited conduction of electrical charge carriers by adjusting junction areas with electrodes in the conventional insulator thin-film containing uniformly distributed defect states.

With this strategy, herein, we have confirmed that electrical charge carriers are stably transported in relatively thick conventional insulator films in MIM structures by controlling an electrode contact area. The electrical currents due to the flow of charge carriers through insulator films flow bi-directionally in MIM structures, and the bi-directional vertical currents are changed to uni-directional vertical currents by applying an oxide semiconductor film as a top electrode in the MIM structures. Moreover, we reveal that the vertical currents through relatively thick insulator films depend on a space charge-limited conduction (SCLC) mechanism which is a type of the bulk-limited conduction mechanisms. Depending on the SCLC mechanism of the vertical currents, a direction, a magnitude, and a rectification ratio of the vertical current in the metal/insulator/oxide semiconductor (MIOS) structures are precisely controlled by electrical properties of the top oxide semiconductor electrode.

## Results

### Unconventional vertical current in a MIOS structure

**Figure 1:**
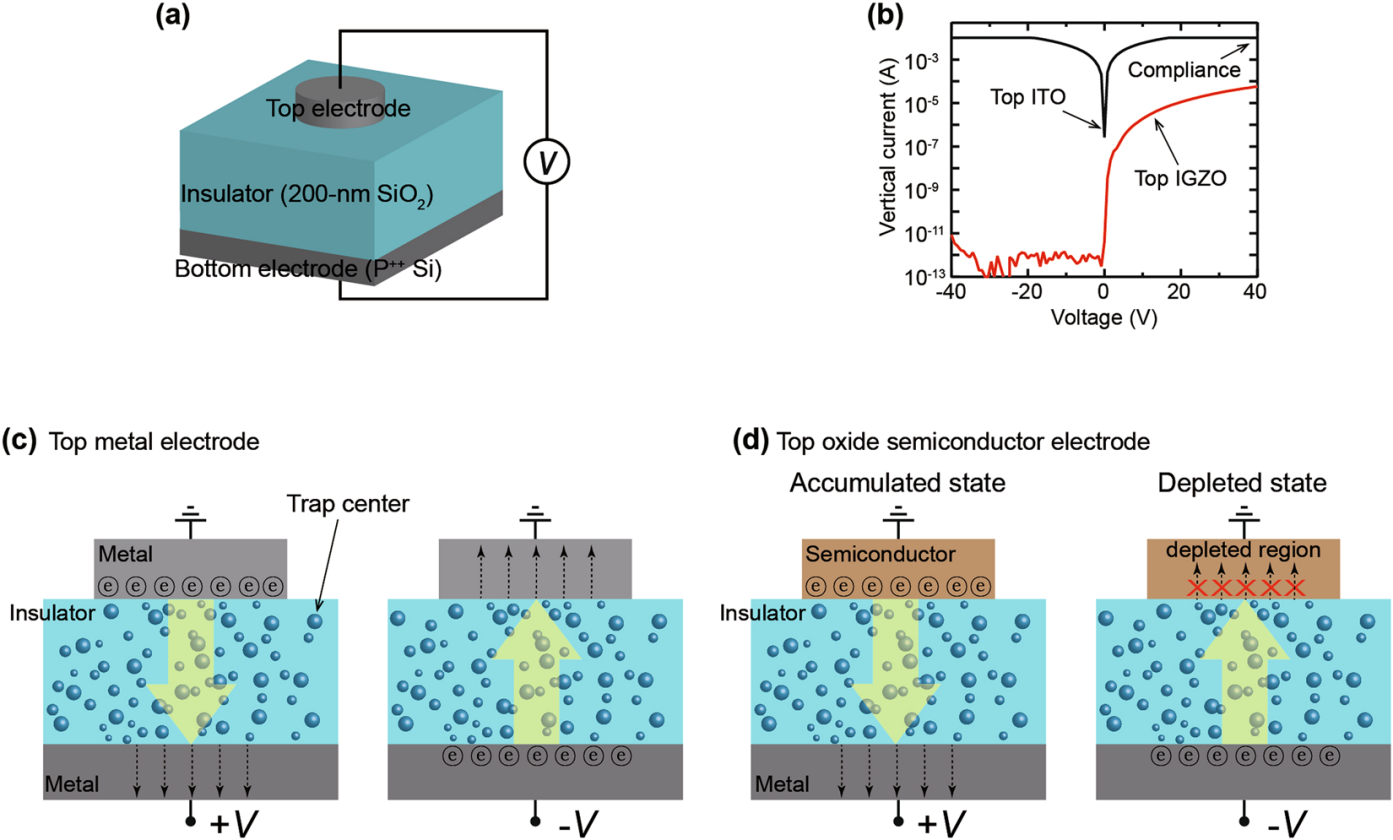
Unconventional vertical current in bottom electrode/200-nm thick insulator/top electrode structure. (**a**) Schematic image of the device consisting of bottom electrode (P^++^ Si)/thick insulator (200-nm SiO_2_)/top electrode. (**b**) The vertical current (I)-voltage (V) characteristics of five devices for each top ITO metal and IGZO semiconductor electrode. (**c**,**d**) The schematic images illustrate that the transporting mechanisms of electrons in (**c**) a metal/insulator /metal (MIM) structure and (**d**) a metal/insulator/oxide semiconductor (MIOS) structure.

To investigate the transport mechanism, we fabricated model devices that consisted of a bottom electrode/insulator/top electrode structure and characterized the output currents at the top electrode in response to an applied voltage to the bottom electrode (Fig. 1a). A heavily doped p-type Si (P^++^ Si) was used as the bottom electrode and a substrate, a silicon dioxide (SiO_2_) with a thickness of 200 nm was mainly used as the insulator, and metal and various oxide semiconductor thin-films with a thickness of 20 nm were used as the top electrode. All of the top electrodes were isolated from the edges of the device using a metal shadow mask to avoid unintended side-contacts with the bottom electrode. First, we investigated the influence of the junction size on the current flowing vertically in a MIM device consisting of a P^++^ Si/200-nm SiO_2_/Al electrode structure. The area of the Al thin-film with a circle shape was varied from 0.79 mm^2^ to 28.26 mm^2^ (diameters from 1 mm to 6 mm). The SiO_2_ film has an inherently good insulating property at a junction size of 0.79 mm^2^, but the current flowing through the 200-nm SiO_2_ film increases sharply as the area of the top Al electrode increases (Supplementary Figure S1). It should be noted that the 200-nm SiO_2_ film maintains a robust pristine insulating property, but the amount of current through the 200-nm SiO_2_ increases due to the size of the top electrode. Consequently, the conventional 200-nm SiO_2_ insulator film serves as a charge transport layer through which electrical charge carriers pass without losing its inherent insulating ability. The phenomenon for which the current increases due to the increase of the electrode area has been consistently reported in MIM structures and RS devices, which is understood to be because the superposition of the trap state in which electrons can move is proportional to the junction region^14–18^. The main focus of this study is to investigate the effect of the top electrode on the current in the MIM structure; therefore, the junction size of all the devices investigated in this work is kept constant at 28.26 mm^2^ (circle with a diameter of 6 mm), which allows the most stable current flow.

In MIM devices using a 20-nm thick indium tin oxide (ITO) metal as the top electrode, a current of 10^−2^ A, which is due to the compliance of our measurement system, flows stably in both directions through the 200-nm SiO_2_. On the other hand, in MIOS devices using a 20-nm thick indium gallium zinc oxide (IGZO) semiconductor thin-film as the top electrode, the vertical current stably flows under a positive voltage, but the current hardly flows and maintains an insulating level of ~10^−12^ A under a negative voltage (Fig. 1b). The current (*I*)-voltage (*V*) characteristics show that the magnitude and direction of the vertical current is highly dependent on the top electrode material: the vertical current flows bi-directionally in MIM device using a top metal electrode; the vertical current flows uni-directionally in MIOS device using a top semiconductor electrode. The 200-nm SiO_2_ layer which is contacted with the top ITO metal electrode can transport the electrons injected from contacts. The electrons can enter from both the top and bottom electrodes into the SiO_2_ film in the MIM device with the ITO electrode; thus, the electrical vertical current flows bi-directionally (Fig. 1c). In the MIOS device with the IGZO semiconductor electrode, the electrons are injected through an electron-accumulated region at the IGZO/SiO_2_ interface by the positive voltage applied to the bottom electrode so that the vertical current level is dependent on the electron conductivity of the top semiconductor electrode and is lower than the device with the top ITO electrode. In contrast, the electrons that are transferred through the SiO_2_ layer from the bottom electrode in response to a negative voltage applied to the bottom electrode are blocked by the electron-depleted region, which serves as a capacitor, at the IGZO/SiO_2_ interface; thus, the current hardly flows and the vertical current is clearly rectified at negative biases (Fig. 1d). The results indicate that the direction and magnitude of the vertical current are controlled by the interface states of the top electrode material and the 200-nm SiO_2_ film serves stable transporting states for the injected electrons.

In most RS devices, charge carriers move through a conducting filamentary path formed in the dielectric thin-film, and therefore, the initial electroforming, which is the set process of forming the conducting filament by dielectric breakdown, should be performed. After the set process, the conducting filament connects both the top and bottom electrodes resulting in a low resistance state. Then, the conducting path is disconnected by an applied reset voltage and the RS device is changed to a high resistance state. The set and reset operations change the connecting state of the filament and leads to a transition between the low and high resistance states. For this reason, the *I*-*V* curves of the RS devices exhibit a large variation at the regime of the set and reset voltages. In contrast, the electroforming process is not necessary in the devices of this work, and the *I*-*V* curves exhibit little variation between the forward and reverse voltage sweeps. The *I*-*V* curves for the MIM devices with the ITO metal electrode show little deviation between both the forward and reverse voltage sweeps (Supplementary Figure S2a). Additionally, regardless of the sweep direction, the MIOS devices using the IGZO semiconductor electrode enable a high current under only the positive voltage range and definitely block the current under the negative voltage regime, furthermore, there is little hysteresis of the *I*-*V* curves between the forward and reverse voltage sweeps (Supplementary Figure S2b). Consequently, the conduction mechanism of the charge carriers passing through the thick insulator film in this study is distinctly different from that of the conventional resistive switching device. In addition, the current conduction mechanism of the MIOS device is also distinct from a conventional Schottky diode. The Schottky diode rectifies the forward current and the reverse current by controlling height of the Schottky junction barrier between the semiconductor and the metal, but the current in the MIOS device is rectified by the interface charge state of the semiconductor in contact with the insulator film. Furthermore, the Schottky diode is made of a semiconductor/metal junction without an insulator, whereas the MIOS device has a crucial difference that a thick insulator acts as a charge transport film between the semiconductor and the metal (Supplementary Figure S3).

### Exploring the film and interface quality

**Figure 2:**
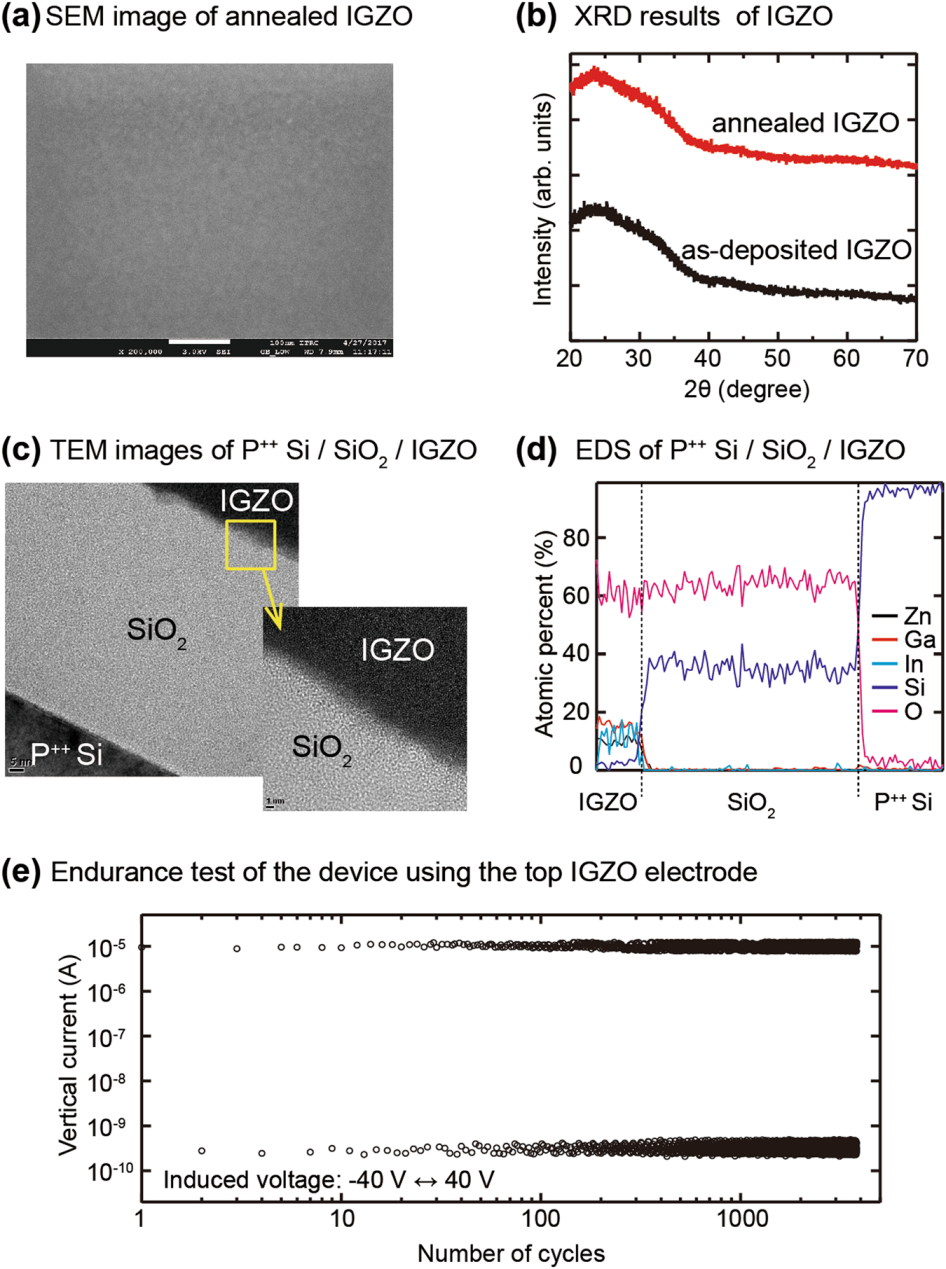
Analysis of the film qualities of the IGZO thin-films and the interface between SiO_2_ and IGZO. (**a**) Top view image of an annealed IGZO thin-film observed using a FESEM. (**b**) X-ray diffraction patterns of the annealed and as-deposited IGZO thin-films. (**c**) A cross-sectional HRTEM image of the P^++^ Si/200-nm SiO_2_/20-nm IGZO structure. (**d**) An EDS line scanning profile of the P^++^ Si/200-nm SiO_2_/20-nm IGZO structure. (**e**) The endurance test result for the MIOS device using the IGZO electrode.

The film quality of the 20-nm IGZO thin-film and the interface between the IGZO and SiO_2_ layers were analyzed after repeating operation of 3,000 cycles to check any damage or deterioration during fabrication processes or operation of the device. A topographical image of an annealed IGZO thin-film observed by a field emission scanning electron microscopy (FESEM) indicates that the film has a small grain size, and X-ray diffractive peaks of the annealed and as-deposited IGZO thin-films prove that the IGZO films have amorphous crystalline structures (Fig. 2a,b). Cross-sectional high resolution transmission electron microscopy (HRTEM) images and energy dispersive spectroscopy (EDS) line scanning profiles of P^++^ Si/200-nm SiO_2_/20-nm IGZO structure show a clear interface of IGZO/SiO_2_ without any inter-mixing layer or atomic diffusion despite thermal annealing process of 350 °C for 90 seconds and repeated operation (Fig. 2c,d). Furthermore, to verify whether flow of the vertical current is caused by any dielectric breakdown due to an applied electric field, an endurance test was carried out for the MIOS device for 3,813 operating cycles. The recorded currents at 40 V and −40 V manifest a reliable flow of current of ~1 × 10^−5^ A and ~5 × 10^−10^ A, respectively, for the repeated cycles. The results mean the 200-nm SiO_2_ layer maintains an excellent insulating property without any dielectric breakdown or insulating degradation (Fig. 2e). Consequently, the vertical currents are not caused by any severe leakage path of the insulator film, and the electrode-limited conduction mechanisms like a quantum tunneling effect of charge carriers is almost impossible because the thickness of the SiO_2_ insulator is 200 nm^19–23^. Instead, it can be reasonably inferred that the electrons are injected and transported through inherent trap centers that randomly exist in the SiO_2_ layer because the vertical current starts to flow near 0 V without a strong external electric field and flows more stably as the junction size increases.

### Temperature dependence of the vertical current

**Figure 3:**
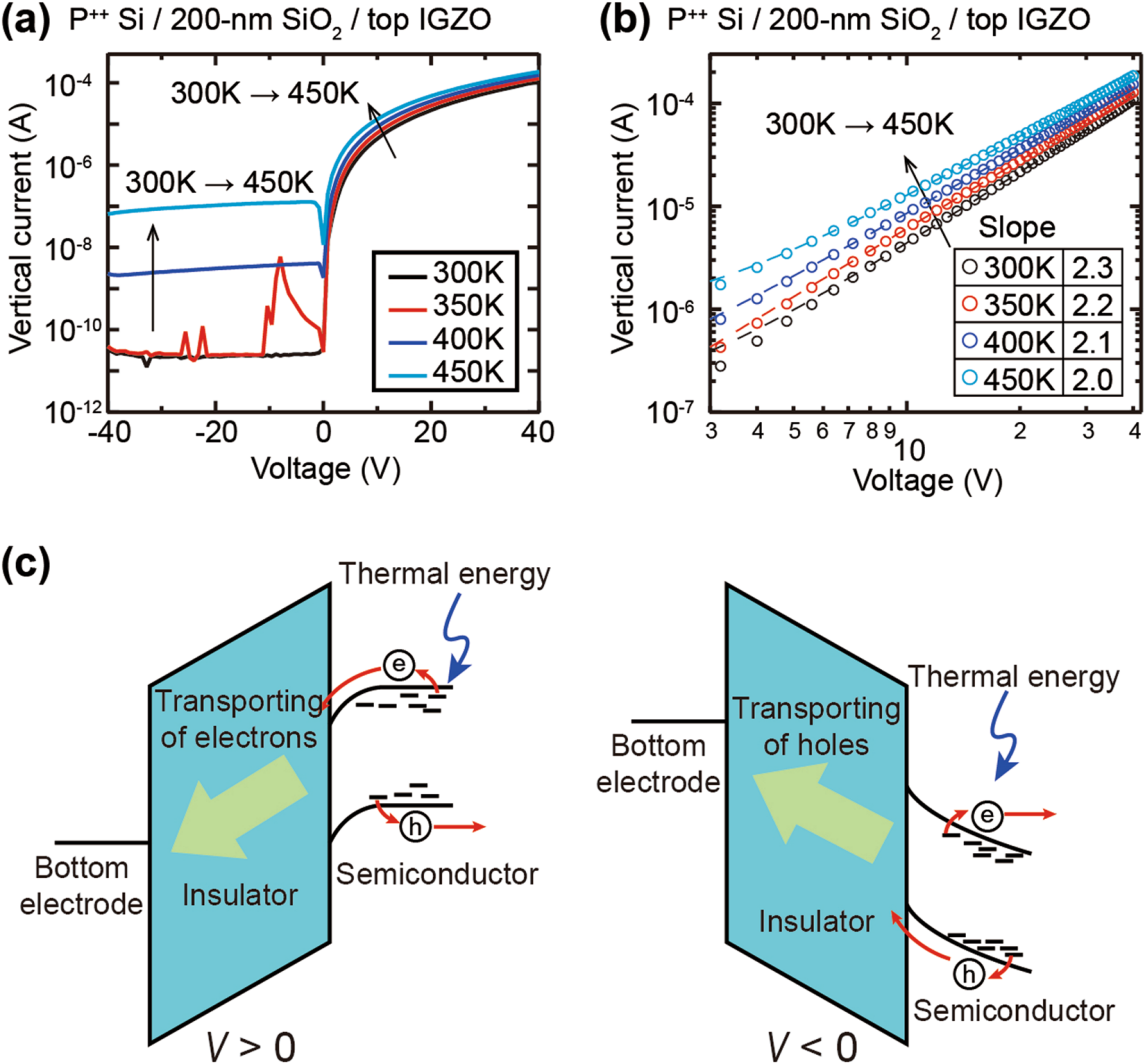
Temperature dependence of the vertical current in the MIOS structure. (**a**) The current increases at both negative and positive voltage ranges as the temperature rises in the MIOS device. (**b**) The *I*-*V* curves of the MIOS device are plotted linearly in log-log axes and the slopes of the curves are around 2.0. (**c**) The schematic images depict that the excited charge carriers in the IGZO layer by thermal energy contribute to the current rise.

The electrical current behavior with temperature changes indicates a conduction mechanism for carriers in many electric devices^24^. In the P^++^ Si/200-nm SiO_2_/20-nm IGZO structure, the uni-directional vertical current increases steadily under positive voltage ranges as the temperature increases from room temperature to 450 K (Fig. 3a). The *I*-*V* curves for all temperatures are linearly fitted in log-log axes, which signify that the conductions of the carriers follow a bulk-limited conduction such as an ohmic or a space charge-limited conduction (Fig. 3b). However, because the current by the ohmic-like conduction decreases by the temperature rise, the conduction of the MIOS device is inconsistent with ohmic conduction. The slopes of all the fitted lines are around 2.0, and there is no transition of the slopes so that the conductions of the charge carriers through the 200-nm SiO_2_ layer evidently are dependent on the space charge-limited conduction (Fig. 3b)^24,25^. Meanwhile, in the MIOS structure, the vertical current under a negative voltage, which hardly flows at room temperature, also begins to increase sharply from 400 K, reaching ~10^−7^ A at 450 K (Fig. 3a). The increase of the currents by temperature at both negative and positive biases can be clearly explained by excited charge carriers in the IGZO electrode. The charge carriers, electrons and holes, are easily excited by the absorbed thermal energy on the tail-states of the top IGZO semiconductor electrode^26,27^, and the excited electrons (or holes) enter into the 200-nm SiO_2_ layer by the positive (or negative) voltages (Fig. 3c). Therefore, it can be ascertained that the current level is highly dependent on the concentration of the charge carriers injected from the contacts, which is consistent with the basic theory that the space charge-limited current is mainly controlled by carriers injected from the electrodes^28^.

### Effect of conductivity of the top semiconductor electrode on the current behavior

**Figure 4:**
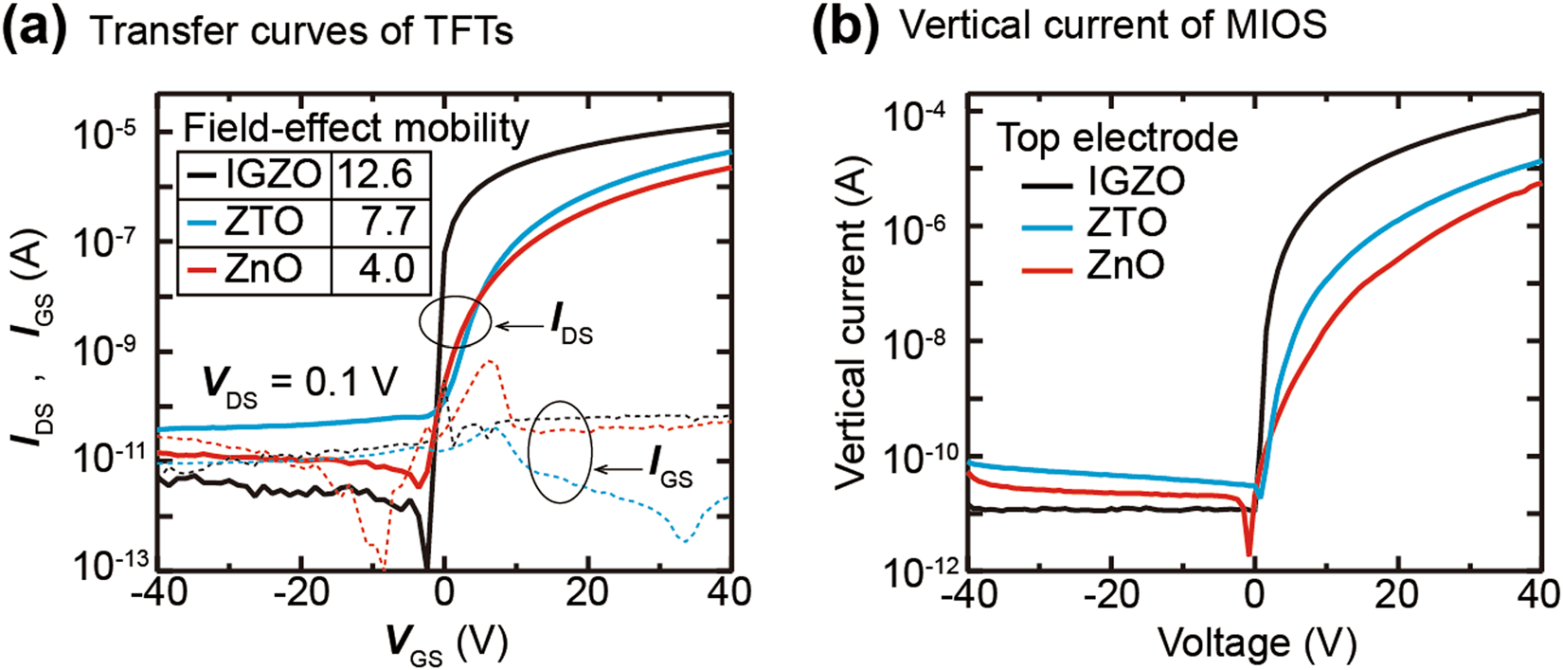
Effect of the electron mobility of the top semiconductor electrode on the vertical current. (**a**) The source-drain currents measured in thin-film transistors (TFTs) using 20-nm IGZO, ZTO and ZnO thin-films as an active layer. (**b**) The vertical current values for the MIOS structures using 20-nm IGZO, ZTO and ZnO thin-films.

The vertical current in the MIOS device using the top semiconductor electrode depends mainly on the concentration of charge carriers injected from the top semiconductor electrode; thus, the current level can be adjusted by controlling the electron mobility or doping level of the top semiconductor material. To explore the effect of the electron mobility on the current, three n-type metal oxide semiconductor materials, IGZO, zinc tin oxide (ZTO) and ZnO, were compared as the top electrode on the P^++^ Si/200-nm SiO_2_ substrate. The thickness of the semiconductor films was constant as 20 nm. We fabricated thin-film transistors (TFTs) using the semiconductor thin-films as an active layer to compare the field-effect mobility of electrons for each 20-nm semiconductor thin-film. The current characteristics between a source and a drain electrode (*I*_DS_), which are measured for a source to drain voltage (*V*_DS_) of 0.1 V, exhibit the typical transfer curves of the n-type oxide semiconductor TFTs^29–31^. The extracted field-effect mobility for the TFTs using the IGZO, ZTO, and ZnO active layers is 12.6, 7.7, and 4.0 cm^2^V^−1^s^−1^, respectively. The *I*_DS_ of each TFT increases as the electron mobility of the each semiconductor active layer increases because the *I*_DS_ is proportional to the electron mobility of the active layer (Fig. 4a). On the other hand, the vertical current in the MIOS device is also proportional to the electron mobility of the top semiconductor electrode. Thus, the vertical current of the MIOS device using the IGZO is the highest, and the lowest vertical current flows in the MIOS device using the ZnO electrode (Fig. 4b). The semiconductor conductivity dependence of the vertical current is also confirmed in the results of the MIOS device using the as-deposited IGZO top electrode. The IGZO film in the as-deposited state has high subgap defect states near the conduction band minimum, so it exhibits poor conductivity^32^. Therefore, a much lower vertical current flows than the MIOS device using the thermal annealed IGZO electrode (Supplementary Figure S4).

**Figure 5:**
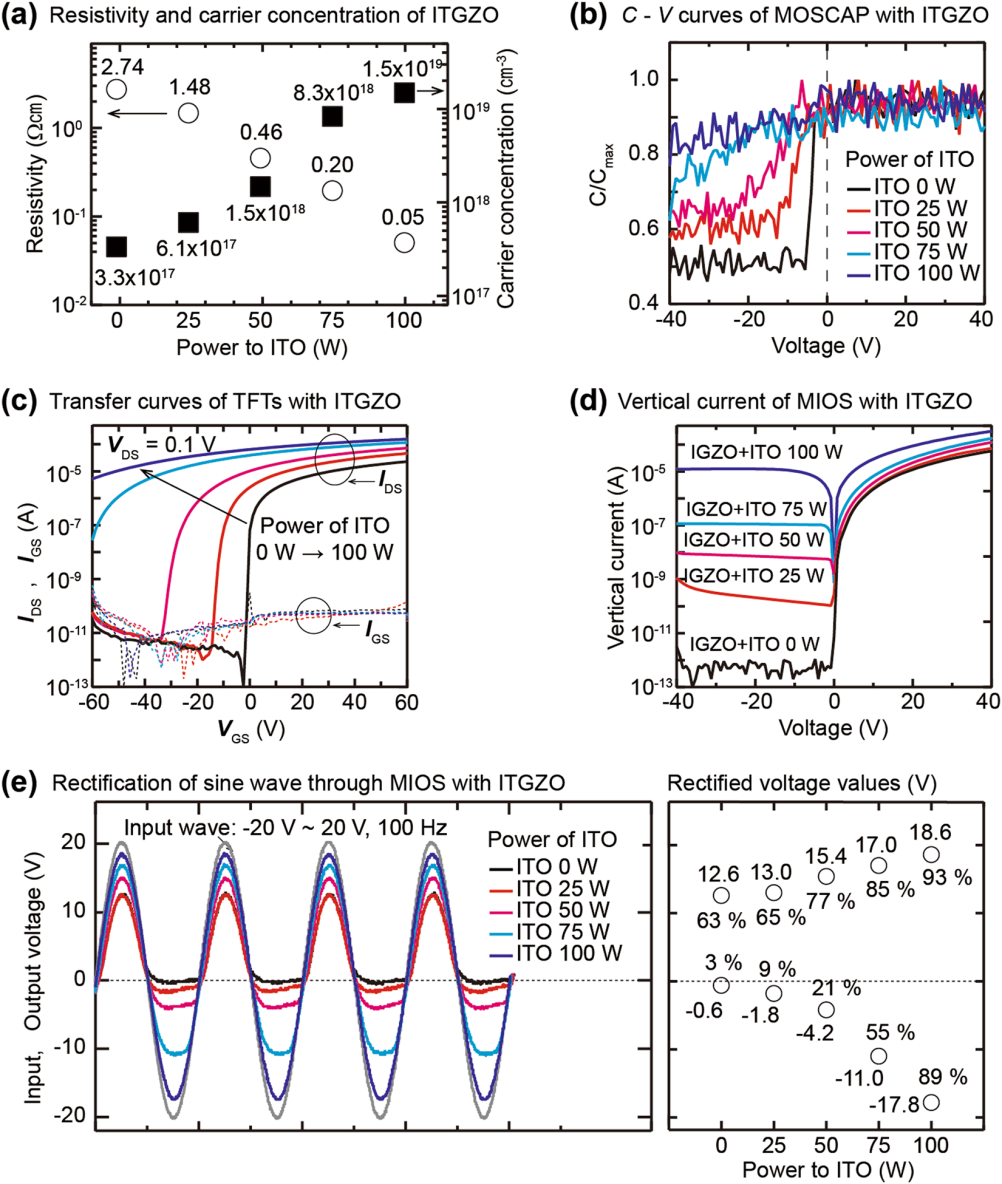
Effect of the doping concentration of the top semiconductor electrode on the current behavior. (**a**) Charge carrier concentration and resistivity of the 20-nm ITO-doped IGZO (ITGZO) thin-films. (**b**) The *C*-*V* characteristics of MOSCAPs using the ITGZO thin-films. (**c**) The source-drain currents measured in thin-film transistors (TFTs) using the ITGZO thin-films as an active layer. (**d**) The vertical current values for the MIOS structures using the 20-nm ITGZO thin-films. (**e**) The output sine waves through the MIOS device of P^++^ Si/200-nm SiO_2_/20-nm ITGZO demonstrate that the direction and rectification ratio of the output waveform are varied by the doping concentration of the top semiconductor electrode.

By the polarity of the applied voltages, the top semiconductor electrode becomes fully electron-accumulated/depleted at the interface with the insulator inducing the uni-directional vertical current. However, it is noted that increasing the doping concentration makes a semiconductor thin-film less depleted at a constant negative voltage. It implies that the vertical current level at the negative voltage can be controlled by the doping level of the top semiconductor electrode. To verify the effect of the doping level on the current direction, the electron concentration of the IGZO film was increased by increasing the content of In in the IGZO thin-film^32^. The In composition of the IGZO thin-film was varied using a co-sputtering method of simultaneously depositing the IGZO and the ITO materials from each IGZO and ITO sputtering target^33^. We increased the content of the In by increasing the power of the ITO target from 0 W to 100 W while keeping the sputtering power of the IGZO target fixed. The high power applied to the ITO target causes more In element to be deposited during the same deposition time, thereby increasing the In content of the IGZO. All compositional ITO-doped IGZO (hereafter denoted as ITGZO) thin-films were deposited to a thickness of 20 nm. From the resistivity values and charge carrier concentrations analyzed by the Hall measurement system, increasing the power applied to the ITO target results in a higher doping state of the ITGZO thin-film enhancing the carrier concentration and lowering the resistivity of the thin-films (Fig. 5a). The The change of the depleted state due to the increased doping was confirmed by the capacitance (*C*)-voltage (*V*) characteristics for metal/oxide/semiconductor capacitors (MOSCAPs) with the ITGZO layers. The continual increase of the *C* value under the negative biases indicates that the depletion region width is substantially extended due to the increased doping (Fig. 5b). The transfer curves (*I*_DS_-*V*_GS_) of the TFTs using the ITGZO layers as the active layer also show that the ITGZO thin-films are partially depleted and are becoming a degenerated semiconductor as the doping level increases (Fig. 5c). In fact, because the characteristic of a semiconductor thin-film approaches the characteristic of a conductor as the doping is increased, the *I*-*V* characteristic of the MIOS device becomes close to that of the MIM device. Therefore, the higher doping level of the top ITGZO electrode in the P^++^ Si/SiO_2_/ITGZO structure enables the higher vertical current at the positive voltage and also causes a significant increase in the vertical current at the negative voltage region (Fig. 5d). These results prove that the polarity and magnitude of the vertical current passing through a thick insulator film are sophisticatedly controlled by the doping concentration of the top semiconductor electrode. To test the potential of the MIOS device whether it can be adopted in real electronic circuits, a sine wave with amplitude of 20 V and a frequency of 100 Hz was applied to the device, and the output waves were measured varying the doping of the top ITGZO electrode. The output wave passing through the MIOS device with the pure IGZO electrode is clearly rectified, enabling a reduced wave of 12.6 V for the positive input sine waves and blocking most of the negative input signals. Moreover, the passed positive output wave steadily increases due to the enhanced conductance of the top ITGZO electrode as the doping level is increased, thereby reaching 93% of the input wave amplitude in the most highly doped device. Furthermore, the amplitude of the rectified negative waveform significantly increases with a higher doping concentration, and the passing ratio of the negative input wave is controlled precisely from 3% to 89% in the regime of the doping levels (Fig. 5e).

## Discussion

In conclusion, we have shown that the vertical current can flow reliably through a thick insulating film of the MIM structure by controlling the junction size. The current is due to the charge carriers passing through the trap centers inherently present in the insulating film and is dominated by the space charge-limited conduction mechanism. The current through the insulating layer in the MIM structure flows bi-directionally, but a MIOS structure with an oxide semiconductor as the top electrode causes the current to flow in only one direction with a high rectification ratio of 5 × 10^6^ at a voltage range between −40 V and 40 V. Because the surface charge of the top oxide semiconductor at the insulator interface changes depending on the polarity of the voltage, the current flows through the fully electron-accumulated surface by the positive voltage, but the fully electron-depleted surface at the negative voltage cuts off the current flow. Then, the vertical current levels can be easily modulated by varying the electron mobility of the top semiconductor electrode, and the rectified signal polarity and rectification ratio of the AC wave passing through the device can be controlled very precisely by the doping level of the top semiconductor electrode. Our new approach to the current flow through insulator films is a simple and universal technique that can be easily used in various electronic devices and is also a prominent solution for such applications as a rectifier, switching device, amplifier, and thin-film diode in oxide electronics.

## Methods

### Deposition of various metal oxide, metal electrodes and insulator thin-films

All of the metal oxide films with a thickness of 20 nm, IGZO, ZTO, ZnO and ITO were sputtered by a RF magnetron sputtering system using an indium gallium zinc oxide (In:Ga:Zn:O = 1:1:1:4 at%), a zinc tin oxide (ZnO:SnO_2_ = 2:3 mol%), a zinc oxide (ZnO) and an indium tin oxide (In_2_O_3_:SnO_2_ = 9:1 wt%) targets, respectively, under 10^−6^ Torr at room temperature. The ITO-doped IGZO thin-films were co-sputtered using the IGZO and ITO targets simultaneously. The RF sputtering power of the IGZO target was fixed to 90 W and the DC sputtering power of the ITO target was varied from 0 W to 100 W. Thickness of all compositional ITO-doped IGZO thin-films was 20 nm. After deposition process, all of the metal oxide thin-films were annealed at 350 °C for 90 seconds in air using a rapid thermal annealing method. The Al electrode with a thickness of 20 nm was deposited via a vacuum thermal evaporation at 10^−6^ Torr. The 200-nm SiO_2_ layer was grown onto the highly boron-doped p-type silicon (P^++^ Si) by a thermal oxidation process. All of the top electrodes including semiconductor and metal thin-films were patterned by metal shadow masks in order to avoid unexpected side-contacts with the bottom electrode.

### Preparation of substrate

The highly boron-doped p-type Si (P^++^ Si) wafers were sequentially cleaned with detergent, de-ionized water, acetone, and isopropyl alcohol. And the size of all specimens was 2 × 2 cm^2^.

### Fabrications of the vertically operated devices

The top Al electrodes were patterned using circle-shaped metal shadow masks with diameters from 0.5 mm to 6 mm. The top electrodes of all devices except the Al top electrode were patterned using a circular metal shadow mask with a diameter of 6 mm. The thickness of all top electrodes was fixed to 20 nm.

### Fabrications of the TFT devices

The TFTs have a bottom gate structure with the P^++^ Si (gate)/200-nm SiO_2_ (gate insulator)/20-nm IGZO (active)/100-nm Al (source and drain). The oxide semiconductor active layers were annealed before the source and drain process. The width and length of the channels in the TFTs are 1000 μm and 50 μm. The active layers were patterned with the same size as the source and drain electrodes in order to block the source-gate leakage current, which begins to flow through the gate insulator film when the active layer area exceeds 12.56 mm^2^. The source-drain voltage was fixed to 0.1 V, and all of the transfer curves were measured at room temperature in a dark.

### Fabrications of the MOS capacitors

The metal/oxide/semiconductor capacitors were fabricated with P^++^ Si/200-nm SiO_2_/20-nm ITO-doped IGZO semiconductors/100-nm Al electrode structure. The area of the semiconductor and Al electrode layers was patterned in the same circle shape with a diameter of 1 mm in order to block the vertical current due to the large area semiconductor layer. The capacitance (*C*) values were measured at a frequency of 20 Hz small signal at room temperature in a dark and each *C* value was normalized by the maximum *C* value.

### Characterization of the fabricated devices

The current-voltage characteristics for all devices were measured using the Agilent 4155B semiconductor parameter analyzer in a dark. The capacitance-voltage curves were analyzed using the Agilent 4284 A precision LCR meter in a dark. The sine wave with a frequency of 100 Hz and amplitude of 20 V was input by the Agilent 33500B waveform generator and a voltage amplifier, and the output voltages were detected by the Tektronix DPO-2024 oscilloscope.

### Data availability

The datasets generated during and/or analyzed during the current study are available from the corresponding author on reasonable request.

## Electronic supplementary material

Supporting Information
