# Supplementary material for: Vertical Transport Control of Electrical Charge Carriers in Insulator/Oxide Semiconductor Hetero-structure
Source: Sci Rep. 2018 Apr 4;8:5643. doi: 10.1038/s41598-018-23990-3 (PMC5884772; doi:10.1038/s41598-018-23990-3)
Supplement: Supplementary file 1 — Supporting Information [file 41598_2018_23990_MOESM1_ESM.docx]

**Supplementary Information**

**Vertical Transport Control of Electrical Charge Carriers in Insulator / Oxide Semiconductor Hetero-structure**

**Jinwon Lee^1,2^, Kapsoo Yoon^3^, Keon-Hee Lim^2^, Jun-Woo Park^2^, Donggun Lee^2^, Nam-Kwang Cho^2^, and Youn Sang Kim^2,4*^**

^1^Samsung Display Company, Ltd, 181 Samsung-ro, Tangjeong-myeon, Asan-si, Chungcheongnam-Do, Republic of Korea

^2^Program in Nano Science and Technology, Graduate School of Convergence Science and Technology, Seoul National University, 1 Gwanak-ro, Gwanak-gu, Seoul 08826, Republic of Korea

^3^Department of Physics, Hanyang University, 222 Wangsimni-ro, Seongdong-gu, Seoul, 04763, Republic of Korea

^4^Advanced Institute of Convergence Technology, 145 Gwanggyo-ro, Yeongtong-gu, Suwon 16229, Republic of Korea.

^*^Corresponding author; Youn Sang Kim (E-mail: [younskim@snu.ac.kr](mailto:younskim@snu.ac.kr), tel: 82-31-888-9131)

**Note S1. Influence of the junction size on the vertical current in the MIM structure**

In order to investigate the effect of the junction area on the current in the MIM device, the vertical current-voltage characteristics were measured for the MIM device composed of P^++^ Si / 200-nm SiO_2_ / 20-nm Al electrode. And the area of top Al electrode with circle shapes was varied from 0.79 mm^2^ to 28.26 mm^2^ (diameters from 1 mm to 6 mm), and the currents were measured at the Al electrode with a probe contact at room temperature in a dark for five devices of each size (Supplementary Figure S1a). In the device with the area of the top Al electrode is 0.79 mm^2^ (circle with a diameter of 1 mm), the vertical current hardly flows (~ pA levels). However, higher vertical current begins to flow bi-directionally when the area of the Al electrode exceeds 12.56 mm^2^ (circle with a diameter of 4 mm), and the vertical current level increases sharply as the area of the top electrode increases (Supplementary Figure S1b). The values of the vertical current at 1 V indicate that the vertical current level increases abruptly above 10^-4^ A as the area of the top electrode increases to 28.26 mm^2^ (circle with a diameter of 6 mm) (Supplementary Figure S1c). In addition, the current density, which is divided by the junction area, also increases abnormally as the electrode area increases (Supplementary Figure S1d).

**
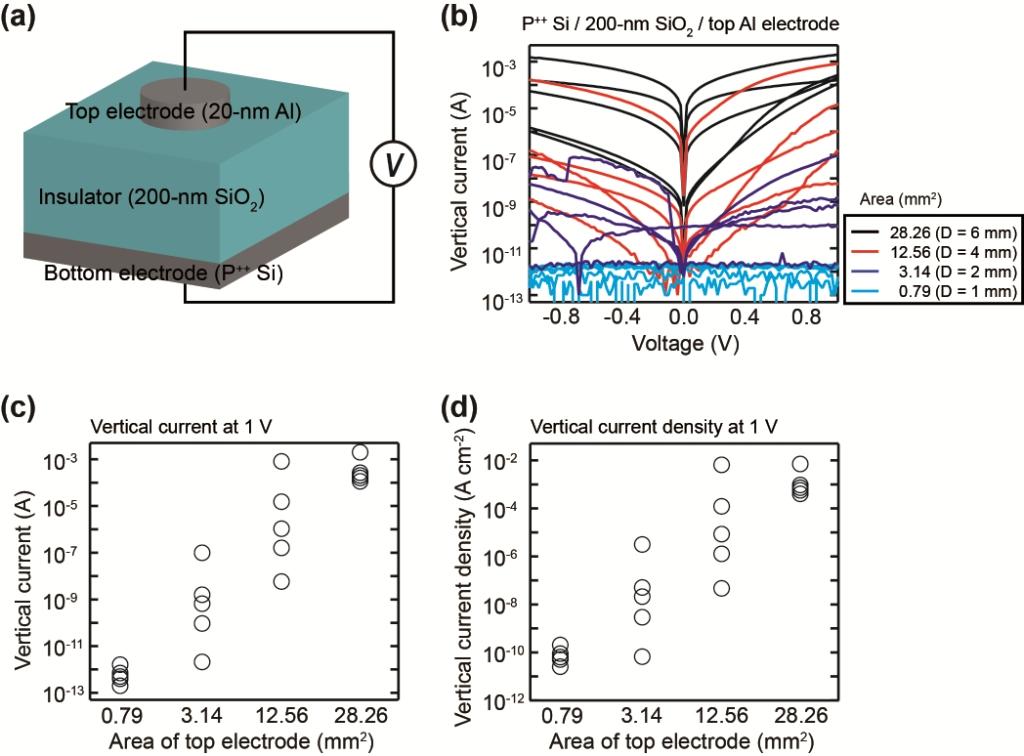
**

**Supplementary Figure S1.** Vertical current of P^++^ Si / 200-nm SiO_2_ / 20-nm Al electrode structure varying area of top Al electrode. (**a**) The schematic image of the MIM device composed of P^++^ Si / 200-nm SiO_2_ / 20-nm Al electrode. (**b**) The vertical current-voltage curves measured for the MIM devices with various area of the Al electrode. (**c**) The values of the vertical current measured at 1 V. (**d**) The vertical current density divided by each area of the Al electrode.

**Note S2. Conduction mechanism through the thick SiO_2_ layer in the MIOS device**

Transport in dielectric or insulator has been extensively studied for many years, and the conduction mechanisms have been well established.^1,2^ The conduction mechanisms are classified by the factors that control the movement of charge carriers through the dielectric. The electrical current through the dielectric may be due to a number of different conduction mechanisms with different dependencies on temperature and electrical bias. Therefore, measuring current density (*J*)-voltage (*V*) characteristics at various temperatures is a very effective technique for identifying the conduction mechanism. In conduction mechanisms, some mechanisms mainly rely on the electrical property in dielectric material. These conductions are called as bulk-limited conduction mechanisms, and include Poole-Frenkel emission, Ionic conduction, Ohmic conduction, and Space charge-limited conduction (SCLC).^3,4^ Among the bulk-limited conduction mechanisms, the SCLC is dominated by the density of electrons injected from the contacts of Ohmic junctions. In the theory of the SCLC, the electrons are entered into the dielectric, and when the trap sites in dielectric fill up, space charges accumulates in dielectric. In the subsequent very strong injection of electrons, all trap sites are filled and the electrons move freely in the dielectric, then the electrical current is completely governed by the space charge. The current governed by the space charge follows the Child’s raw where the current is proportional to the square of the voltage. Thus, the current by the SCLC is theoretically expressed as:

$J_{SCLC}=\frac{9}{8}\mu\varepsilon\frac{V^{2}}{d^{3}}$ (1)

where *J*_SCLC_ is the current density due to space charge-limited conduction, *μ* is the mobility of electron, *ε* is the relative permittivity of the dielectric insulator, *V* is the electric potential, and *d* is the thickness of the dielectric. Thus, the space charge-limited current is proportional to the square of the voltage. When the vertical *I*-*V* characteristics in the MIOS devices are plotted on log-log axis, the *I*-*V* curves exhibit a linear relationship and the slopes of the fitted lines are around 2.0 in Figure 3b; consequently, it can be rationally concluded that the vertical current in our devices follows the space charge-limited conduction mechanism.


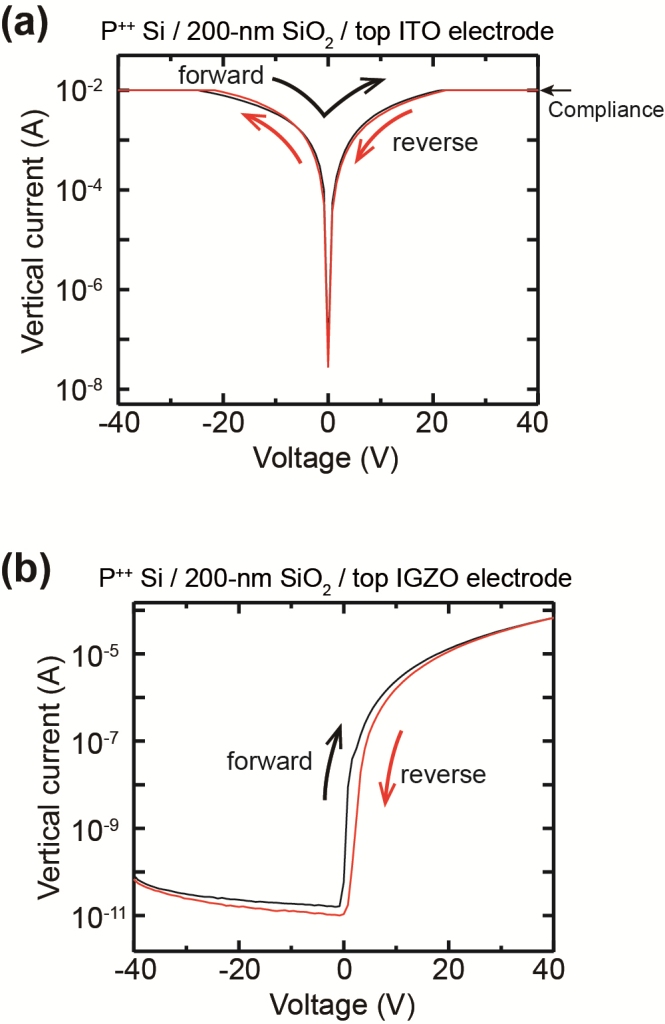


**Supplementary Figure S2.** The vertical current characteristics measured by forward sweep from – 40 V to 40 V and reverse sweep from 40 V to – 40 V. (**a**) The vertical current flows bi-directionally in the P^++^ Si / SiO_2_ / top ITO metal electrode devices and reaches 10^-2^ A, which is the compliance value of the *I*-*V* measuring system at 20 V. (**b**) The vertical current flows uni-directionally under only positive voltage ranges in the MIS devices using top IGZO semiconductor electrode, and the current values under negative biases are equivalent to the insulating states. (**a**, **b**) The *I*-*V* characteristics measured by forward sweep from -40 V to 40 V and reverse sweep from 40 V to -40 V are equivalent without large variations.


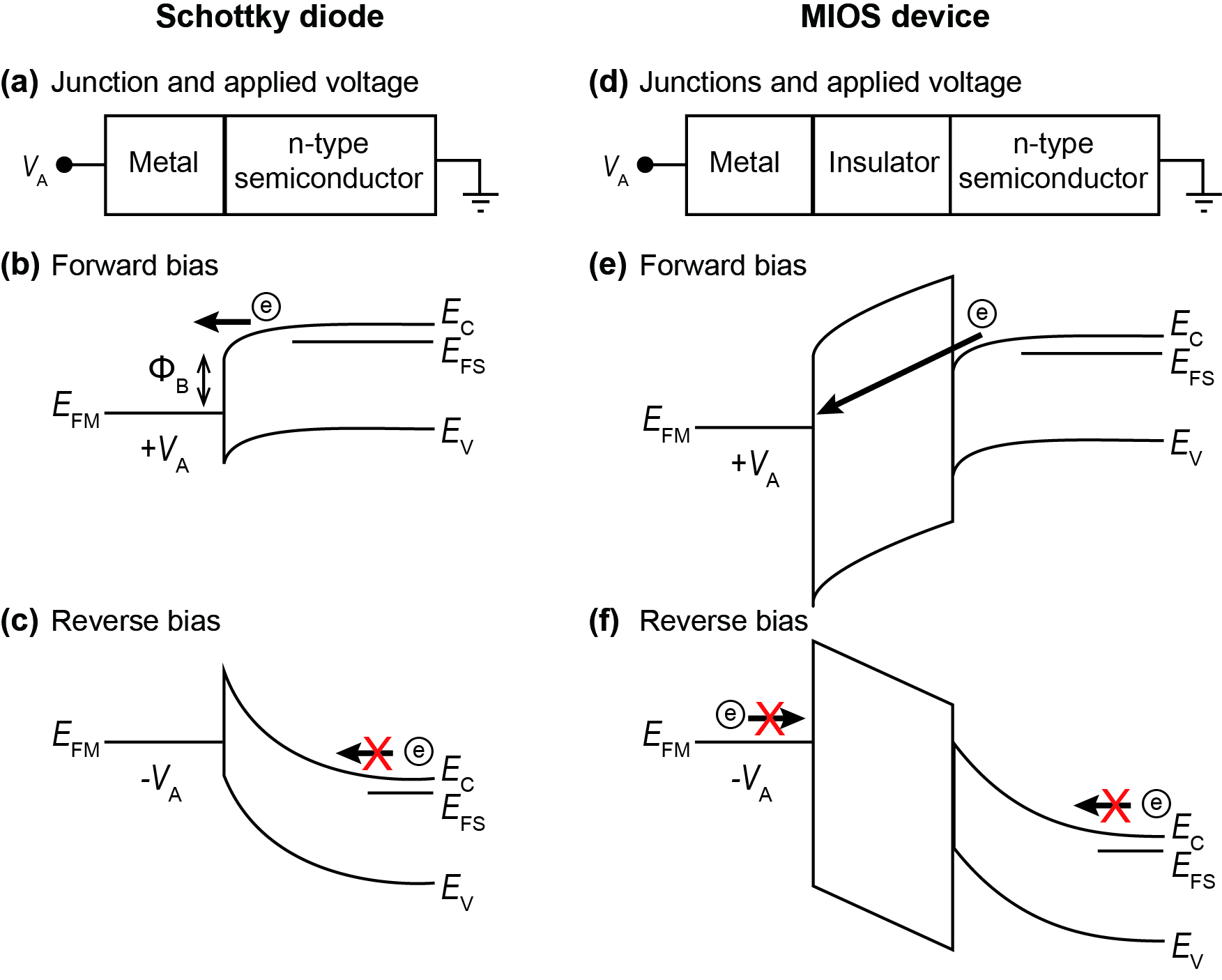


**Supplementary Figure S3.** Comparison of the Schottky diode and the MIOS device operation. (**a-c**) Schematics of Schottky diode. (**a**) Device junction and voltage polarity. (**b**) Energy band diagram schematic under forward bias of *V*_A_ > 0. (**c**) Energy band diagram schematic under reverse bias of *V*_A_ < 0. (**d-f**) Schematics of MIOS device. (**d**) Device junctions and voltage polarity. (**e**) Energy band diagram schematic under forward bias. (**f**) Energy band diagram schematic under reverse bias.


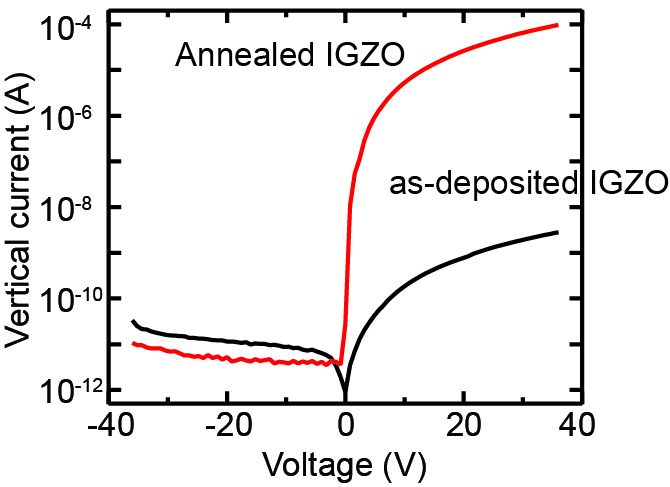


**Supplementary Figure S4.** Variation of vertical current behavior with IGZO film annealing effect.

**REFERENCES**

1. Bentarzi, H. *Transport in Metal-Oxide-Semiconductor Structures*, Springer, Berlin (2011).

2. Lampert M. A. Simplified theory of space-charge-limited currents in an insulator with traps. *Phys. Rev*. **103**, 1648–1656 (1956).

3. Chiu, F. -C. A review on conduction mechanisms in dielectric films. *Adv. Mater. Sci. Eng*. **2014**, (2014).

4. Cheong, K. Y. *et al*. Current conduction mechanisms in atomic-layer-deposited HfO_2_/nitride SiO_2_ stacked gate on 4H silicon carbide. *J. Appl. Phys*. **103**, 084113 (2008).
